# Risk of eating disorders and the relationship with interest in modern culture among young female students in a university in Bangladesh: a cross-sectional study

**DOI:** 10.1186/s12905-023-02186-6

**Published:** 2023-01-25

**Authors:** Md Monjurul Ahasan, Md Shahidul Quadir Patwari, Masahiro Yamaguchi

**Affiliations:** 1 Department of Physiology, Kochi Medical School, Kochi University, Kohasu, Okocho, Nankoku, Kochi 783-8505 Japan; 2 Dhaka International University, Road #1, Block #F, Banani, Dhaka 1213 Bangladesh

**Keywords:** Eating disorders, Female, Bangladesh, Modern culture, Fashionable clothes, Indian TV shows

## Abstract

**Background:**

Eating disorders (EDs) emerge most often in adolescent girls. While the basic neural and psychiatric mechanisms of ED development remain unknown, their incidence has increased with sociocultural modernization. To determine what aspects of modern culture are related to EDs, we examined the relationship between predisposition to EDs and interest in several modern cultural factors among young female university students in Bangladesh.

**Methods:**

A cross-sectional study was conducted in a population of 196 female students aged 18–29 years in a university in Bangladesh. Their predisposition to EDs was examined using the Eating Attitudes Test-26 (EAT-26), and their interest in modern culture was evaluated by grading interest in fashionable clothes, Indian TV shows/serials, luxury food and social network activities, and by examining involvement in cultural activities such as playing, dancing, parties and singing. The relationship between predisposition to EDs and interest in modern culture was then examined. The relation between EAT-26 score and body mass index (BMI) was also examined.

**Results:**

The EAT-26 revealed that 37% of the participants were at risk of developing an ED. Correlation analyses showed that a high EAT-26 score was correlated with a high interest in fashionable clothes and Indian TV shows/serials, but not with a high interest in luxury food or social network activities, or involvement in cultural activities, such as playing, dancing, parties and singing. Further, EAT-26 questions in the dieting category and oral control category, but not the bulimia and food preoccupation category, were correlated with interest in cultural factors. EAT-26 score showed no significant correlation with BMI.

**Conclusions:**

The results of this study indicate that the risk of EDs is related to an interest in several facets of modern culture. Monitoring the cultural interests of adolescent females, who are continually exposed to modern culture and highly susceptible to EDs, will contribute to the prevention of EDs.

**Supplementary Information:**

The online version contains supplementary material available at 10.1186/s12905-023-02186-6.

## Background

Eating disorders (EDs) are characterized by abnormal eating behaviors, which typically emerge in females during or prior to adolescence [1, 2]. Patients with anorexia nervosa, characterized by restricted food intake and severe weight loss, and bulimia nervosa, characterized by binge eating followed by purging, aim to achieve or maintain a low body weight due to distorted images about their body shape and weight [3]. The lifetime prevalence rates of anorexia nervosa and bulimia nervosa are up to 4% and 3%, respectively, in Western countries [4]. Mortality risk is elevated five times or more in ED patients [4].

The incidence of EDs has increased continually over the past several decades worldwide including Asia, in parallel with the modernization of society that is promoted by industrialization and urbanization [5–8]. Industrialization and urbanization lead to many cultural changes, including nutritional status, food palatability, interest in and ideals of beauty, degree of exposure to social media and gender roles, and the increasing prevalence of EDs has been related to such modern cultures [9–11]. In particular, young girls, who are most susceptible to EDs, are continually exposed to such effects of rapidly changing industrialization and urbanization [12], which is also applicable to South Asia [13, 14]. Bangladesh has recently experienced significant economic growth and modernization, and a substantial proportion of young people in Bangladesh are at risk of developing EDs [7, 15, 16]. To understand the mechanisms underlying the development of EDs in young women, and to prevent its occurrence, it is important to understand what aspects of modern culture are related to the predisposition to EDs.

The risk of EDs has been evaluated using the Eating Attitudes Test-26 (EAT-26) questionnaire, which consists of 26 questions related to dieting, bulimia and preoccupation with food, and oral control [17]. This test has been applied successfully to young women in Bangladesh to gain insight into their ED risk [15, 16]. To examine the correlation between the ED risk and cultural factors of young women in Bangladesh, we recruited female students from a university in Bangladesh and conducted both the EAT-26 and an analysis of several features of modernization.

Participants’ interest in luxurious food and fine dining was chosen as a representative aspect of modernized food culture. Interest in fashionable clothes was examined because image of beauty and body image disturbances are considered to underlie EDs [18–20]. Interest in Indian TV shows was examined because many young people in Bangladesh follow the trends of modern Indian culture via such shows [21]. Interest in Facebook was examined because there has been a rapid increase in the use of social media among young people in Bangladesh as the means of communication and sharing of modern culture. Involvement in cultural activities such as singing and dancing was also examined because many young university students enjoy such activities in and outside the university.

## Methods

### Participants

A total of 208 women were initially recruited from the three campuses of Dhaka International University (Green Road campus, Banani campus, and Satarkul permanent campus). Exclusion criteria were the age over 30 (n = 5) and incomplete answers to the questionnaire (n = 7). As a result, 196 participants aged 18–29 years (mean ± standard deviation [SD] range: 22.2 ± 1.9 years) were subjected to analysis. The sample size was determined using G*Power 3.1 software based on a power of 80%, significance level of 0.05, and a medium effect size of 0.25, to detect differences among four groups with different levels of interest in cultural factors, which gave a required total sample size of 180.

### Ethical considerations

Orientation sessions for the study were conducted by teachers and other faculty members regarding the purpose, procedure, maintenance of confidentiality and anonymity, and limited usage of the data for research. After the explanation, all participants provided informed consent orally. Almost all of the potential subjects agreed to participate in the study.

### Questionnaire

The survey was conducted between March and July 2019. After informed consent had been obtained, the EAT-26 survey was administered by providing written questionnaire forms. The validity and reliability of EAT-26 in Bangladesh had been shown previously [15, 16]. Students also completed a questionnaire on age, height, and body weight, as well as interest in fashionable clothes, luxurious food and fine dining, Facebook, and Indian TV shows/serials. The response options were “strong interest,” “moderate interest,” “little interest,” and “no interest.” The questionnaire also asked about involvement in cultural activities (playing, dancing, parties, singing, and others). For this particular question, participants could check any number of choices (0–5 among 5 choices).

### Sampling technics and quality assurance

All the questionnaires were provided in the university classrooms. Several tens of participants in a classroom were delivered with written questionnaire forms and filled in answers, supervised by teachers or faculty staffs who gave instructions and responded to technical questions from participants. After collecting the questionnaire forms with answers, incomplete forms having blank columns were excluded from analysis.

### EAT-26

The EAT-26 was administered according to standard methods [17]. The questionnaire consists of 26 items in three categories: dieting, bulimia and food preoccupation, and oral control. A 6-point scale with responses ranging from “always” to “never” was used. For questions 1–25, scores of 3, 2, 1, 0, 0, and 0 corresponded to “always,” “usually,” “often,” “sometimes,” “rarely,” and “never,” respectively; the order was reversed for question 26. Total score ranges from 0 to 78. Scores < 20 correspond to “no risk,” 20–49 to “at risk,” and 50–78 to “consistent with EDs.”

### Body mass index (BMI)

BMI was calculated by dividing the weight in kilograms by the square of height in meters (kg/m^2^) [22]. BMI was categorized according to the World Health Organization’s guidelines [23] as follows: < 18.4, underweight (< 16, severely underweight; 16.0–16.9, moderately underweight; and 17.0–18.4, slightly underweight); 18.5–24.9, normal (18.5–22.9, normal-low; and 23.0–24.9, normal-high); and ≤ 25.0, overweight (25.0–29.9, pre-obese; and ≥ 30, obese).

### Statistical analysis

The association between BMI and EAT-26 score was determined by calculating Pearson’s correlation coefficient (*r*). The relationships of EAT-26 total and subscale scores with interest in modern culture were analyzed by Spearman’s rank evaluation test and a one-way analysis of variance (ANOVA) with the Tukey–Kramer post hoc test. The relationship between ED risk groups (“no risk” and “at risk” groups) and the level of interest in modern culture was assessed by the chi-square test. A multivariate logistic regression analysis was further conducted to adjust for possible interrelations among interest in different cultural factors. The relationships between the scores for each question on the EAT-26 and interest in modern culture were analyzed by Spearman’s rank evaluation test and the Kruskal–Wallis test. In all analyses, *P* < 0.05 was taken to indicate statistical significance. Logistic regression analysis was performed using JMP software (version 10; SAS Institute Inc., Cary, NC, USA). All other analyses were performed using IBM SPSS Statistics software (version 23.0; IBM Corp., Armonk, NY, USA). All graphs were constructed using GraphPad Prism software (version 8.0; GraphPad Software Inc., La Jolla, CA, USA).

## Results

### Analysis of EAT-26 scores

**Fig. 1:**
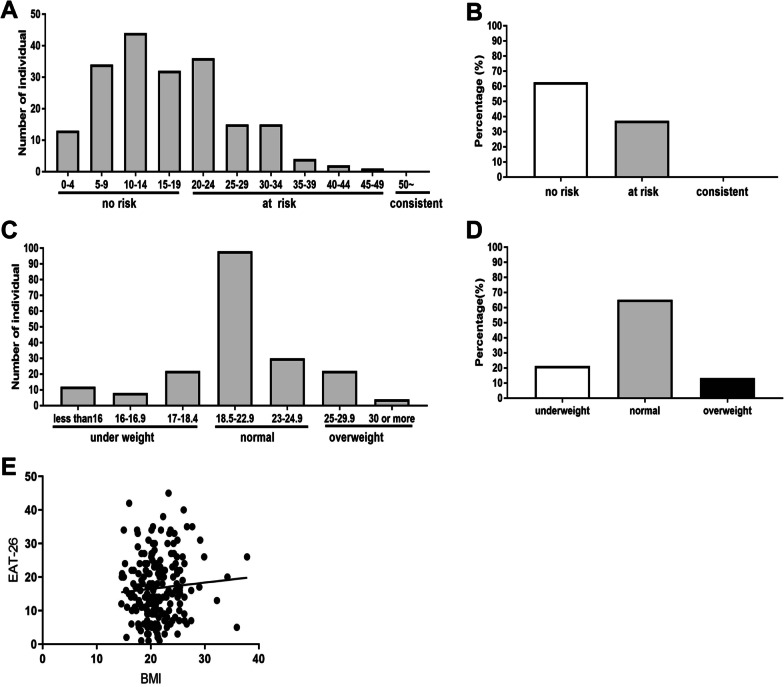
EAT-26 score and BMI. **A** Distribution of the EAT-26 scores of all participants. **B** Relative proportions of participants classified as “no risk,” “at risk,” and “consistent with EDs,” corresponding to EAT-26 scores < 20, 20–49, and ≥ 50, respectively. No participants were classified into the latter group. **C** BMI distribution of all participants. **D** Relative proportions of underweight, normal, and overweight participants according to the WHO’s criteria. **E** Results of Pearson’s correlation analysis between BMI and EAT-26 score (*r* = 0.076, *P* = 0.29)

A total of 196 female university students aged 18–29 years participated in this study, consisting of 49.5% aged 21–23 years, 26.5% aged 24–26 years, 21.9% aged 18–20 years, and 2.0% aged 27–29 years. Therefore, the majority (> 70%) were in the age range 18–23 years. Figure 1A shows the distribution of EAT-26 total scores, which ranged between 1 and 49. According to the EAT-26 cutoffs described above [17], 63% of participants with scores < 20 were categorized as “no risk,” while 37% scoring between 20 and 49 were categorized as “at risk.” No participants had a score ≥ 50, which is considered “consistent with EDs” (Fig. 1B). The score distribution was similar to that in previous studies in Bangladesh [16], which confirmed that a substantial percentage of female university students in Bangladesh are at risk of developing EDs.

BMI ranged between 14.6 and 37.8 (Fig. 1C). According to the cutoffs for Asian countries [23], 21.4% of participants were underweight (BMI < 18.5), 63.8% were of normal weight (BMI = 18.5–24.9), and 14.8% were overweight (BMI ≥ 25.0) (Fig. 1D). There was no significant correlation between BMI and EAT-26 total score (*r* = 0.076, *P* = 0.29) (Fig. 1E).

### Correlations between cultural factors and EAT-26 scores

Levels of interest in fashionable clothes, luxury food/fine dining, Indian TV shows, Facebook, and cultural activities (e.g., dancing, partying, and singing) were analyzed as representative aspects of modern culture. The numbers of participants and percentages of total participants with strong, moderate, little, and no interest in fashionable clothes were 56 (28.6%), 58 (29.6%), 62 (31.6%), and 20 (10.2%), respectively; those for luxury food/fine dining were 48 (24.5%), 58 (29.6%), 64 (32.7%), and 26 (13.3%), respectively; those for Indian TV shows were 10 (5.1%), 21 (10.7%), 52 (26.5%), and 113 (57.7%), respectively; and those for Facebook were 38 (19.4%), 72 (36.7%), 66 (33.7%), and 20 (10.2%), respectively. The numbers of participants who had taken part in playing, dancing, partying, and singing were 36 (18.4%), 34 (17.3%), 19 (9.7%), and 48 (24.5%), respectively; 68 (34.7%) selected “other” activities.

**Fig. 2:**
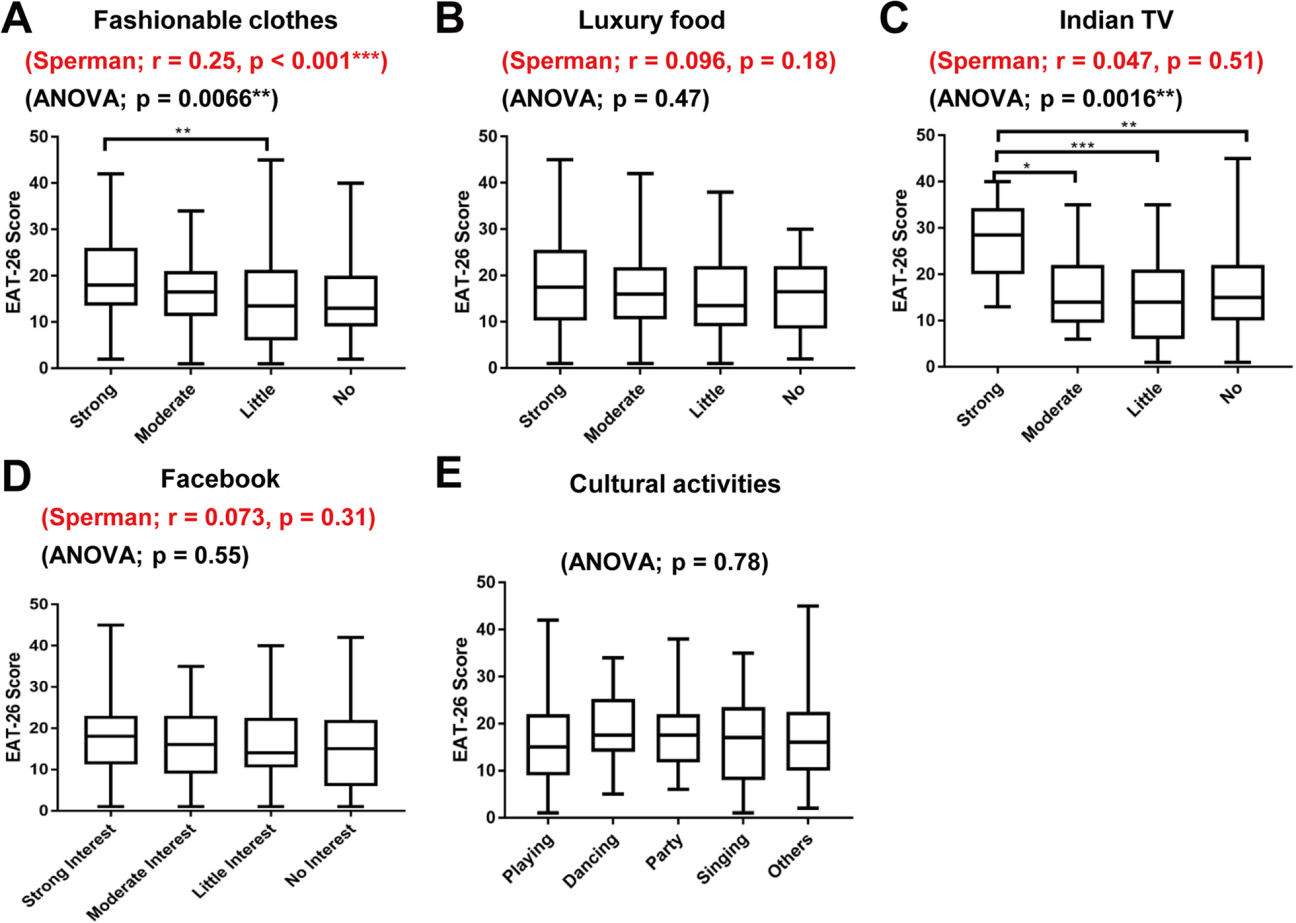
Relationships between interest in aspects of modern culture and EAT-26 score. The relationships of interest in fashionable clothes (**A**), luxury food/fine dining (**B**), Indian TV shows/serials (**C**), Facebook (**D**), and participation in cultural activities (**E**) with EAT-26 scores are shown. Boxes indicate the 25th and 75th percentiles, whiskers denote the minimum and maximum values, and lines inside the boxes indicate median values. The *r*- and *P*-values for Spearman’s rank evaluation are shown in red, and the *P*-values for the one-way ANOVA are shown in black. Positive *r* values in Spearman’s rank evaluation indicate a correlation of higher interest in modern culture with higher EAT-26 scores. *P*-values for the Turkey-Kramer post hoc tests are shown for each pairwise comparison (*P < 0.05; **P < 0.01; and ***P < 0.001)

Next, we examined whether interest in the above aspects of modern culture were correlated with EAT-26 scores (Fig. 2). The overall correlation between interest in modern culture and EAT-26 score was evaluated by Spearman’s rank evaluation test, and the differences in EAT-26 scores among groups with different degrees of interest in modern culture were evaluated by a one-way ANOVA with post hoc multiple comparisons.

Interest in fashionable clothes was significantly correlated with EAT-26 score, with a higher level of interest correlating with a higher EAT-26 score (*r* = 0.25, *P* < 0.001; Spearman’s rank evaluation test) (Fig. 2A). The positive values for Spearman’s correlation coefficient in this study indicate that a higher level of interest in modern culture was correlated with higher EAT-26 score. EAT-26 scores were not significantly correlated with interest in other aspects of modern culture, including luxury food, Indian TV shows, and Facebook (Fig. 2B–D).

**Fig. 3:**
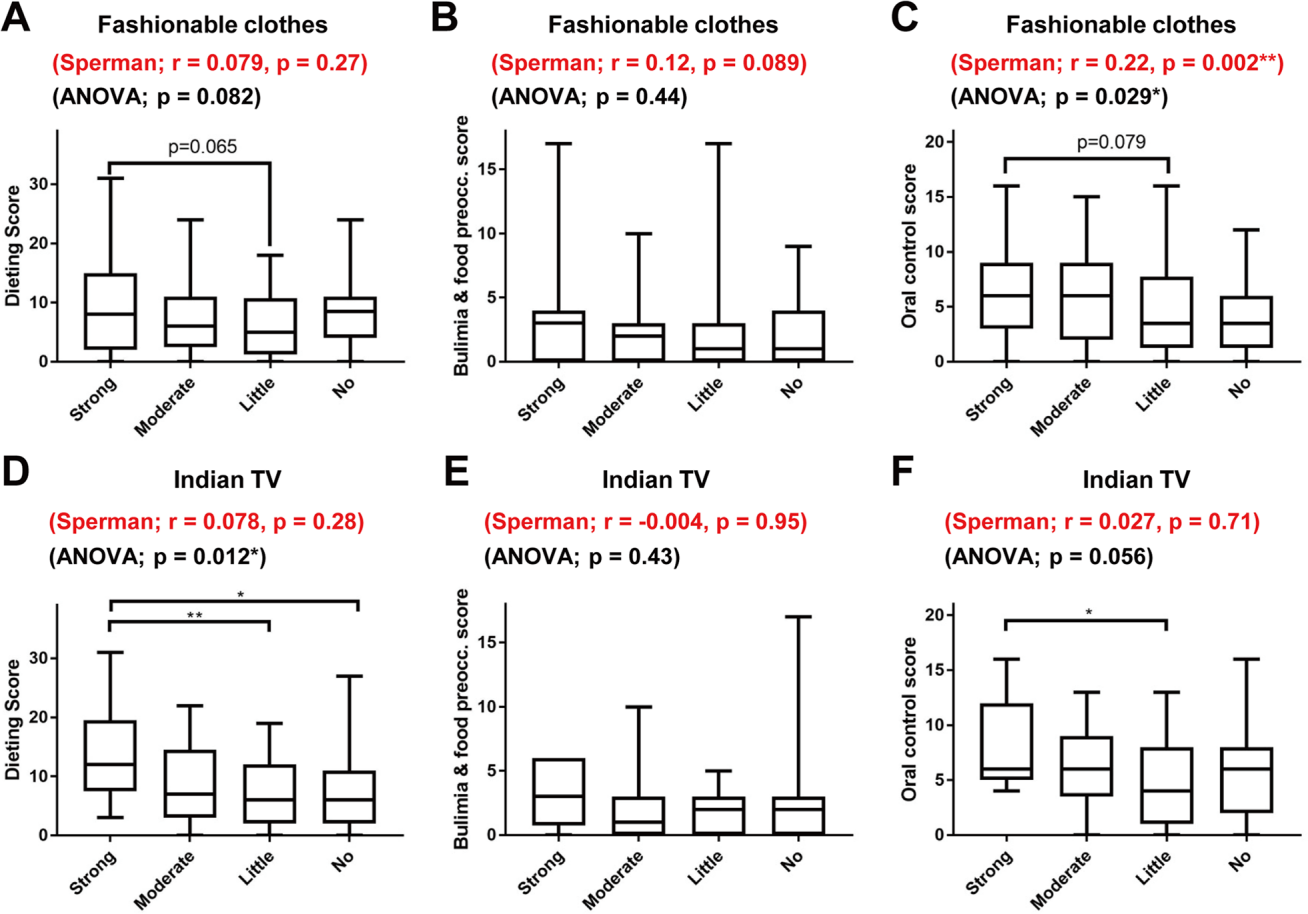
Relationships between interest in aspects of modern culture and EAT-26 subscale scores. The relationships of interest in fashionable clothes (**A**–**C**) and Indian TV shows (**D**–**F**) with dieting (**A**, **D**), bulimia and food preoccupation (**B**, **E**), and oral control (**C**, **F**) EAT-26 subscale scores are shown. Boxes indicate the 25th and 75th percentiles, whiskers denote the minimum and maximum values, and lines inside the boxes indicate median values. The *r* and *P*-values for Spearman’s rank evaluation are shown in red, and the *P*-values for one-way ANOVA are shown in black. Positive *r* values in Spearman’s rank evaluation indicate a correlation between higher level of interest in modern culture with higher EAT-26 subscale score. *P*-values for the Turkey-Kramer post hoc test are shown for each pairwise comparison (*P < 0.05 and **P < 0.01)

Next, we examined differences among groups according to the above data. There was a significant difference in EAT-26 score between groups with different levels of interest in fashionable clothes (*P* = 0.0066; one-way ANOVA) and Indian TV shows (*P* = 0.0016) (Fig. 3A and C). Post hoc analyses showed that participants with a strong interest in fashionable clothes had higher EAT-26 scores than those with little interest (Fig. 3A). Participants with a strong interest in Indian TV shows had higher EAT-26 scores than those with moderate, little, or no interest (Fig. 3C). Interest in luxury food/fine dining (*P* = 0.47), Facebook (*P* = 0.55), and involvement in cultural activities (*P* = 0.78) was not significantly correlated with EAT-26 score (Fig. 2B, D, and E).

We also classified participants into “at risk” and “no risk” groups according to the EAT-26 cutoffs outlined above, and examined correlations with interest in modern culture. Interest in Indian TV shows was significantly correlated with ED risk (*P* = 0.032; chi-square test) (Table 1). There were no significant correlations of the other four cultural factors with ED risk, whereas participants with a strong interest in fashionable clothes showed a higher rate of ED risk (48.2%) than those with less interest (30.6–35.0%). A multivariate logistic regression analysis was performed to adjust for possible interrelations among interests in different cultural factors. For this analysis, participants with no/little interest were classified as a low interest group, and those with moderate/high interest were classified as a high interest group. With regard to the involvement in cultural activities, participants involved in playing, dancing, partying, or singing were grouped together (*n* = 121) and compared with the group of participants not involved in these activities (*n* = 75). The results showed a significant association for interest in Indian TV (*P* = 0.030) with an odds ratio of 2.48 (95% confidence interval, 1.09–5.74; Table 2). There were no significant associations of the other four cultural factors with ED risk. Thus, the overall patterns of univariate and multivariate analyses according to the EAT-26 cutoffs were similar.

**Table 1:** Correlation between interest in modern culture and ED risk

EAT score	Strong	Moderate	Little	No	χ^2^
Fashionable clothes					
20–49	27	20	19	7	χ^2^ = 4.271, 3, *p* = 0.234
1–19	29	38	43	13	
ED risk (%)	48.2	34.5	30.6	35.0	
Luxury food/glittering restaurant					
20–49	19	21	23	10	χ^2^ = 0.202, 3, *p* = 0.977
1–19	29	37	41	16	
ED risk (%)	39.6	36.2	35.9	38.5	
Indian TV serials					
20–49	8	8	16	41	χ^2^ = 8.805, 3, *p* = 0.032*
1–19	2	13	36	72	
ED risk (%)	80.0	38.1	30.8	36.3	
Facebook					
20–49	17	22	27	7	χ^2^ = 2.713, 3, *p* = 0.438
1–19	21	50	39	13	
ED risk (%)	44.7	30.6	40.9	35.0	

EAT score	Playing	Dancing	Party	Singing	Others	χ^2^
Cultural activities						
20–49	13	12	8	20	24	*χ*^2^ = 0.680, 4, p = 0.954
1–19	23	19	11	28	44	
ED risk (%)	36.1	38.7	42.1	41.7	35.3	

Participants are classified into "at risk" (EAT score: 20—49) and "no risk" (EAT score: 1—19) groups.

Number of participants with different grades of interest for individual modern cultures is indicated.

ED risk (%) indicates percentage of participants scored 20-49 among all participants scored 1-49.

**Table 2:** Analysis of association of interest in modern culture with ED risk by multivariate logistic regression

Variables	OR (95% CI)	*p*-value
Fashionable clothes^a^	1.54 (0.80, 3.03)	0.201
Luxury fiood / glittering restaurant^a^	0.86 (0.50, 1.80)	0.860
Indian TV serials^a^	2.48 (1.09, 5.74)	0.030*
Facebook^a^	0.67 (0.36, 1.23)	0.196
Cultural activities^b^	1.40 (0.75, 2.64)	0.288

^a^Low interest group (no/little interest) vs. high interest group (moderate/high interest)

^b^Group not involved in playing, dancing, parties or singing vs. group involved either in the four cultural activities

OR, odds ratio; CI, confidence interval

Taken together, these analyses showed that young female students with higher levels of interest in fashionable clothes and those with a stronger interest in Indian TV tended to have higher EAT-26 scores and a higher ED risk.

### Correlation between cultural factors and EAT-26 subscale scores for question categories

The 26 questions in EAT-26 are classified into the dieting category (13 questions), bulimia and food preoccupation category (6 questions), and oral control category (7 questions) (Table 3) [17]. First, we examined which EAT-26 subscale scores for these categories were correlated with interest in fashionable clothes and Indian TV shows (Fig. 3). A significant correlation was observed between interest in fashionable clothes and oral control subscale score (*r* = 0.22, *P* = 0.002; Spearman’s rank evaluation test), while there were no significant correlations for other combinations (Fig. 3).

**Table 3:** Correlation between cultural factors and the scores for each question of EAT-26

Category	Q. no	Questions	Fashionable clothes		Indian TV	
			Sperman's rank	Kruskal–Wallis	Sperman's rank	Kruskal–Wallis
Dieting	1	Am terrified about being overweight	*p* = 0.015*	*p* < 0.001***	*p* = 0.880	*p* = 0.550
	6	Aware of the calorie content of foods that I eat	*p* = 0.188	*p* = 0.357	*p* = 0.184	*p* = 0.610
	7	Particularly avoid food with a high carbohydrate content (i.e. bread, rice, potatoes)	*p* = 0.171	*p* = 0.236	*p* = 0.495	*p* = 0.710
	8	Feel extremely guilty after eating	*p* = 0.400	*p* = 0.700	*p* = 0.948	*p* = 0.032*
	11	Am preoccupied with a desire to be thinner	*p* = 0.553	*p* = 0.053	*p* = 0.883	*p* = 0.844
	12	Think about burning up calories when I exercise	*p* = 0.006**	*p* = 0.047*	*p* = 0.853	*p* = 0.009**
	14	Am occupied with the thought of having fat on my body	*p* = 0.227	*p* = 0.089	*p* = 0.540	*p* = 0.117
	16	Avoid foods with sugar in them	*p* = 0.439	*p* = 0.831	*p* = 0.149	*p* = 0.031*
	17	Eat diet foods	*p* = 0.418	*p* = 0.748	*p* = 0.919	*p* = 0.740
	22	Feel uncomfortable after eating sweets	*p* = 0.507	*p* = 0.697	*p* = 0.262	*p* = 0.150
	23	Engage in dieting behavior	*p* = 0.183	*p* = 0.454	*p* = 0.949	*p* = 0.045*
	24	Like my stomach to be empty	*p* = 0.592	*p* = 0.543	*p* = 0.481	*p* = 0.783
	26	Enjoy trying new rich foods	*p* < 0.001***	*p* = 0.001***	*p* = 0.869	*p* = 0.666
Bulimia and food preoccupation	3	Find myself preoccupied with food	*p* = 0.348	*p* = 0.808	*p* < 0.001***	*p* = 0.003**
	4	Have gone on eating binges where I feel that I may not be able to stop	*p* = 0.998	*p* = 0.853	*p* = 0.365	*p* = 0.144
	9	Vomit after I have eaten	*p* = 0.659	*p* = 0.901	*p* = 0.474	*p* = 0.644
	18	Feel that food controls my life	*p* = 0.162	*p* = 0.498	*p* = 0.883	*p* = 0.464
	21	Give too much time and thought to food	*p* = 0.227	*p* = 0.142	*p* = 0.403	*p* = 0.132
	25	Have the impulse to vomit after meals	*p* = 0.583	*p* = 0.939	*p* = 0.222	*p* = 0.567
Oral control	2	Avoid eating when I am hungry	*p* = 0.630	*p* = 0.832	*p* = 0.059	*p* = 0.006**
	5	Cut my food into small *p*ieces	*p* = 0.090	*p* = 0.403	*p* = 0.588	*p* = 0.044*
	10	Feel that others would prefer if I ate more	*p* = 0.341	*p* = 0.134	*p* = 0.199	*p* = 0.775
	13	Other people think that I am too thin	*p* = 0.014*	*p* = 0.057	*p* = 0.800	*p* = 0.501
	15	Take longer than others to eat my meals	*p* = 0.006**	*p* = 0.053	*p* = 0.815	*p* = 0.636
	19	Display self-control around food	*p* = 0.826	*p* = 0.146	*p* = 0.606	*p* = 0.015*
	20	Feel that others pressure me to eat	*p* = 0.650	*p* = 0.468	*p* = 0.159	*p* = 0.301

*P*-values of Sperman's rank evaluation test and Kruskal–Wallis test are shown

Bold black letters indicate positive correlation, plain black letters negative correlation, and gray letters non-significant values

*p < 0.05; **p < 0.01; ***p < 0.001

Differences between groups were then analyzed. A significant difference in oral control subscale score was observed among the four groups with strong, moderate, little, and no interest in fashionable clothes (*P* = 0.029; one-way ANOVA) (Fig. 3C). Multiple comparisons did not detect significant differences for a particular pair of groups, with the smallest *P*-value between the strong interest and little interest groups (*P* = 0.079). The difference in dieting subscale scores was not statistically significant but had a relatively small *P*-value (*P* = 0.082, one-way ANOVA) (Fig. 3A). Although not significant, multiple comparisons showed the smallest *P*-value between the strong and little interest groups (*P* = 0.065). The difference in the bulimia and food preoccupation subscale scores was not statistically significant (*P* = 0.44, one-way ANOVA) (Fig. 3B).

The dieting subscale scores differed significantly among the four groups with different levels of interest in Indian TV shows (*P* = 0.012, one-way ANOVA) (Fig. 3D). Multiple comparisons showed significant differences between the strong and little interest groups (*P* = 0.008) and between the strong and no interest groups (*P* = 0.011). The difference in oral control subscale scores was not statistically significant, although the *P*-value was relatively small (*P* = 0.056, one-way ANOVA) (Fig. 3F), and multiple comparisons showed a statistically significant difference in the scores between the strong and little interest groups (*P* = 0.048). The difference in the bulimia and food preoccupation subscale scores was not statistically significant (*P* = 0.43, one-way ANOVA) (Fig. 3E).

Overall, the analyses indicate that interest in fashionable clothes and Indian TV shows tended to be correlated with the dieting and oral control EAT-26 subscale scores, where interest in fashionable clothes showed a stronger correlation with the oral control subscale score and interest in Indian TV shows a stronger correlation with the dieting subscale score.

### Correlations between cultural factors and the scores for each question on the EAT-26

We finally examined which questions on the EAT-26 questionnaire were correlated with interest in fashionable clothes and Indian TV shows (Table 3). In Spearman’s rank evaluation correlation analysis, interest in fashionable clothes was positively correlated with two dieting questions (Nos. 1 and 12) and two oral control questions (Nos. 13 and 15). Interest in fashionable clothes was negatively correlated with one dieting question (No. 26). Interest in Indian TV shows showed no positive correlation with any questions and a negative correlation with one bulimia and food preoccupation question (No. 3). Spearman’s r values for the positive correlations are shown in Additional file 1: Fig. S1.

Then, group differences were examined with the Kruskal–Wallis test (Table 3). Among the four groups with different levels of interest in fashionable clothes, significant differences were observed for the scores of two dieting questions (Nos. 1 and 12). Multiple comparison tests showed higher scores in the higher interest groups (Additional file 1: Fig. S1). While significant differences were observed for the scores of one dieting question (No. 26), multiple comparison tests showed an inverse trend in that higher scores were seen in the lower interest groups (data not shown). Among the four groups with different levels of interest in Indian TV shows, significant differences were observed for the scores of three dieting questions (Nos. 12, 16, and 23) and three oral control questions (Nos. 2, 5, and 19). Multiple comparison tests showed higher scores in the higher interest groups (Additional file 1: Fig. S1). While significant differences were also observed for the scores of another dieting question (No. 8) and another bulimia and food preoccupation question (No. 3), multiple comparison tests showed an inverse trend with higher scores in the lower interest groups (data not shown).

These analyses for each question indicate that interest in fashionable clothes and Indian TV shows is positively correlated with particular questions in the dieting and oral control categories of the EAT-26.

## Discussion

The present study shows that a high EAT-26 score was correlated with a high interest in fashionable clothes and Indian TV shows/serials, but not with a high interest in luxury food or social network activities, or involvement in cultural activities, such as playing, dancing, partying, and singing. Specific questions in the EAT-26 dieting and oral control categories, but not the bulimia and food preoccupation category, were correlated with interest in these modern cultural factors. These results indicate that the risk of EDs is related to an interest in several aspects of modern culture.

The incidence of EDs has increased with industrialization and urbanization worldwide, including Asia [5–8]. Consistent with previous studies in Bangladesh, 37% of the female university students (aged 18–29 years; range: 22.2 ± 1.9 years) in our study were classified as at risk of developing EDs. In one previous study, 37.6% of university students (both males and females) aged 17–28 years (range: 21.0 ± 2.5 years) were classified as at risk of EDs [16], and in another study 23.5% of female university students aged 21.02 ± 1.65 years were classified as at risk [15]. Our study confirms that a substantial proportion of young females in Bangladesh are at risk of developing EDs.

Our study shows a correlation between interest in fashionable clothes and ED risk. Adolescent females experience physical and identity-related changes, and acquire a greater awareness both of their own bodies and those of others [24]. This greater body awareness may enhance interest in fashionable clothes. Excess body awareness can develop into a body image disturbance, which is considered both a cause and effect of EDs [18–20]. A body image disturbance is characterized by undue influence of body shape and weight on self-esteem [3], as well as thinking that others judge their worth largely in terms of these physical characteristics. Another aspect of body image disturbance is a discrepancy between the actual and perceived body size. ED patients tend to overestimate their body size in comparison to healthy controls [25]. A strong interest in fashionable clothes may promote concern about body shape and overestimation of body size, both of which increase the risk of EDs.

Our study also revealed a correlation between ED risk and interest in Indian TV shows/serials. TV programs, especially those delivered from India to Bangladesh, convey images of the beauty and body size ideals created by the fashion and beauty industries. Images associated with beauty ideals may contribute to body dissatisfaction, defined as the discrepancy between one’s actual and ideal body shape [11]. In fact, exposure to TV and magazines is correlated with body image dissatisfaction [26, 27].

Interest in fashionable clothes and Indian TV shows were significantly correlated with the dieting and oral control EAT-26 subscale scores in the present study. This is consistent with the notion that body image disturbance and ED development are closely linked [18–20]. Fashionable clothes and Indian TV shows promote beauty and body size ideals and self-awareness of body shape/weight, and dieting and oral control is related to the achievement of an ideal body shape/weight.

An analysis of the scores for each question on the EAT-26 also showed correlations between interest in fashionable clothes/Indian TV shows and dieting/oral control questions. Interestingly, the questions showing significant correlations were different between interest in fashionable clothes and Indian TV shows (see Table 3). While the reason for this difference is unclear, this observation suggests that fashionable clothes and Indian TV shows have different effects on ED risk and that the variety of questions on the EAT-26 is important to understand the relations between cultural factors and ED risk.

Social media platforms, including Facebook, Twitter, Instagram, and YouTube, allow the viewing and sharing of user-generated content. The rapid growth of social media has been shown to be associated with a number of psychiatric disorders, including EDs [28, 29]. A correlation between Facebook usage and body image concerns in adolescent girls has been reported previously [30]. However, our analysis revealed no significant correlation between interest in Facebook and ED risk. Young women in Bangladesh may view more potentially ED-related content, such as thin body ideals, via TV than Facebook. Facebook encourages real-world interactions among friends and groups sharing cultural interests, which may promote healthy lifestyles to a greater extent than other social media platforms [31]. Given the general shift from TV to online media, attention should be paid to the usage of social media by young women to prevent EDs. A limitation of the present study is that the questionnaire did not include objective measures such as the time length spent on Facebook and TV. Degree of involvement in these activities may relate to the risk of EDs, which remains to be addressed in future studies.

In contrast to interest in fashionable clothes and Indian TV shows, our study did not show a relationship between interest in luxury food/fine dining and ED risk. While abnormal food intake is the central symptom of EDs, food avoidance and binge eating/purging are considered to be manifestations of a body image disturbance, rather than causes of EDs [19, 20]. EDs likely develop in association with abnormal learning and memory processes that link food with traumatic experiences related to the thin ideal [32]. Such associations may not have yet manifested in our study population of female university students. Consistent with this suggestion, interest in modern culture showed no significant relationship with bulimia and preoccupation with food, which are directly linked to abnormal eating.

Lifestyles differ among populations even in the same country. Although a positive correlation between EAT-26 total score and BMI was reported in a previous study in Bangladesh [15], our study and that of Pengpid et al. [16] found no such correlation. This may have been due to differences in the proportions of overweight participants between the studies (21.2% in the study of Al Banna et al. [15] vs. 14.8% in our study). Analyses of other populations that include significant numbers of overweight participants are required to obtain a more comprehensive understanding of the relationships between aspects of modern culture and ED risk.

## Conclusion

This study shows that young female university students in Bangladesh with a strong interest in fashionable clothes and Indian TV shows as aspects of modern culture tended to have a greater ED risk, and that these interests were correlated with the dieting and oral control EAT-26 subscale scores, but not with the bulimia and food preoccupation subscale score. These results are consistent with the suggestion that the development of EDs is related to increased body awareness and body image disturbances. Monitoring the cultural interests of adolescent females, who are continually exposed to modern culture and highly susceptible to EDs, may contribute to the prevention of EDs.

## Supplementary Information

**Additional file 1.** Relationships between interest in modern culture and scores for each EAT-26 question.

## Data Availability

The data sets used and analyzed in the present study are available from the corresponding authors on reasonable request.

## Abbreviations

BMI: Body mass index
EAT-26: Eating Attitudes Test-26
EDs: Eating disorders
