# Supplementary material for: Risk of eating disorders and the relationship with interest in modern culture among young female students in a university in Bangladesh: a cross-sectional study
Source: BMC Womens Health. 2023 Jan 25;23:35. doi: 10.1186/s12905-023-02186-6 (PMC9878867; doi:10.1186/s12905-023-02186-6)
Supplement: Supplementary file 1 — Additional file 1. Relationships between interest in modern culture and scores for each EAT-26 question. [file 12905_2023_2186_MOESM1_ESM.pdf]

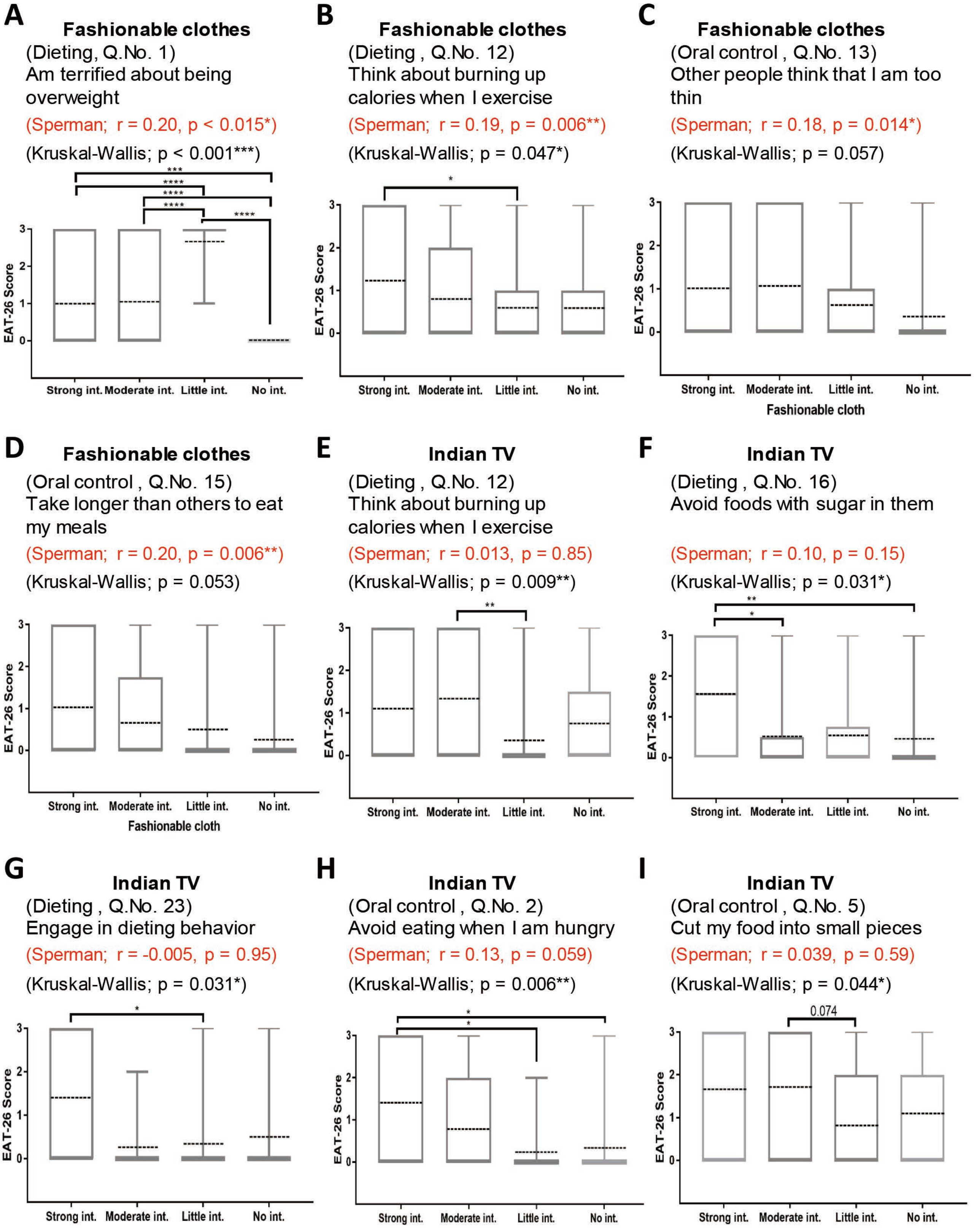
A
Fashionable clothes
(Dieting, Q.No. 1)
Am terrified about being overweight
(Sperman; r = 0.20, p < 0.015*)
(Kruskal-Wallis; p < 0.001***)
***
****
****
****
****
EAT-26 Score
3
2
1
0
Strong int.
Moderate int.
Little int.
No int.
B
Fashionable clothes
(Dieting , Q.No. 12)
Think about burning up calories when I exercise
(Sperman; r = 0.19, p = 0.006**)
(Kruskal-Wallis; p = 0.047*)
*
EAT-26 Score
Strong int.
Moderate int.
Little int.
No int.
C
Fashionable clothes
(Oral control , Q.No. 13)
Other people think that I am too thin
(Sperman; r = 0.18, p = 0.014*)
(Kruskal-Wallis; p = 0.057)
EAT-26 Score
Strong int.
Moderate int.
Little int.
No int.
Fashionable cloth
D
Fashionable clothes
(Oral control , Q.No. 15)
Take longer than others to eat my meals
(Sperman; r = 0.20, p = 0.006**)
(Kruskal-Wallis; p = 0.053)
EAT-26 Score
Strong int.
Moderate int.
Little int.
No int.
Fashionable cloth
E
Indian TV
(Dieting , Q.No. 12)
Think about burning up calories when I exercise
(Sperman; r = 0.013, p = 0.85)
(Kruskal-Wallis; p = 0.009**)
**
EAT-26 Score
Strong int.
Moderate int.
Little int.
No int.
F
Indian TV
(Dieting , Q.No. 16)
Avoid foods with sugar in them
(Sperman; r = 0.10, p = 0.15)
(Kruskal-Wallis; p = 0.031*)
**
*
EAT-26 Score
Strong int.
Moderate int.
Little int.
No int.
G
Indian TV
(Dieting , Q.No. 23)
Engage in dieting behavior
(Sperman; r = -0.005, p = 0.95)
(Kruskal-Wallis; p = 0.031*)
*
EAT-26 Score
Strong int.
Moderate int.
Little int.
No int.
H
Indian TV
(Oral control , Q.No. 2)
Avoid eating when I am hungry
(Sperman; r = 0.13, p = 0.059)
(Kruskal-Wallis; p = 0.006**)
*
*
EAT-26 Score
Strong int.
Moderate int.
Little int.
No int.
I
Indian TV
(Oral control , Q.No. 5)
Cut my food into small pieces
(Sperman; r = 0.039, p = 0.59)
(Kruskal-Wallis; p = 0.044*)
0.074
EAT-26 Score
Strong int.
Moderate int.
Little int.
No int.

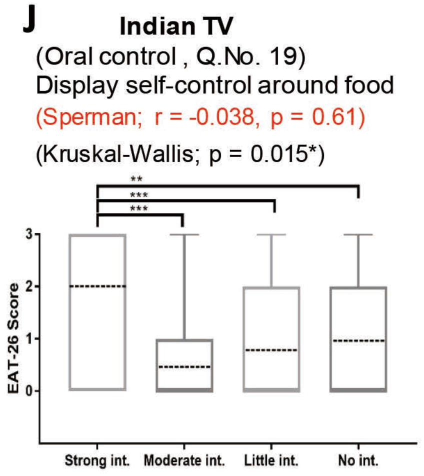

Supplementary Figure 1. Relationship between interest in modern culture and scores for each EAT-26 question

Relationships of interest in fashionable clothes and Indian TV shows with the scores for each EAT-26 question are illustrated by the boxplot. Only significant positive correlations are shown. Boxes indicate the 25th and 75th percentiles, whiskers denote the minimum and maximum values, and dotted lines inside the boxes indicate median values. The r- and p-values for the Sperman's rank evaluation are shown in red, and the p-values for the Kruskal-Wallis test are shown in black. Positive r-values in Sperman's rank evaluation indicate the correlation of higher interest in modern culture with higher scores for each question. P-values for the multiple comparisons with Bonferroni correction are shown for each pairwise comparison (*p < 0.05; **p < 0.01; ***p < 0.001; ****p < 0.0001).
